# A Review of Integrative Omic Approaches for Understanding Rice Salt Response Mechanisms

**DOI:** 10.3390/plants11111430

**Published:** 2022-05-27

**Authors:** Mohammad Asad Ullah, Muhammad-Redha Abdullah-Zawawi, Rabiatul-Adawiah Zainal-Abidin, Noor Liyana Sukiran, Md Imtiaz Uddin, Zamri Zainal

**Affiliations:** 1 Faculty of Science and Technology, Universiti Kebangsaan Malaysia (UKM), Bangi 43600, Malaysia; maullah09@gmail.com (M.A.U.); liyana@ukm.edu.my (N.L.S.); 2 Bangladesh Institute of Nuclear Agriculture (BINA), BAU Campus, Mymensingh 2202, Bangladesh; imtiaz@bina.gov.bd; 3 UKM Medical Molecular Biology Institute (UMBI), Universiti Kebangsaan Malaysia, Kuala Lumpur 56000, Malaysia; mraz@ukm.edu.my; 4 Biotechnology and Nanotechnology Research Centre, Malaysian Agricultural Research and Development Institute (MARDI), Serdang 43400, Malaysia; rabiatul@mardi.gov.my; 5 Institute of System Biology (INBIOSIS), Universiti Kebangsaan Malaysia (UKM), Bangi 43600, Malaysia

**Keywords:** bioinformatics, ion transport, omics, GWAS, transgenic, genome editing, rice, salinity

## Abstract

Soil salinity is one of the most serious environmental challenges, posing a growing threat to agriculture across the world. Soil salinity has a significant impact on rice growth, development, and production. Hence, improving rice varieties’ resistance to salt stress is a viable solution for meeting global food demand. Adaptation to salt stress is a multifaceted process that involves interacting physiological traits, biochemical or metabolic pathways, and molecular mechanisms. The integration of multi-omics approaches contributes to a better understanding of molecular mechanisms as well as the improvement of salt-resistant and tolerant rice varieties. Firstly, we present a thorough review of current knowledge about salt stress effects on rice and mechanisms behind rice salt tolerance and salt stress signalling. This review focuses on the use of multi-omics approaches to improve next-generation rice breeding for salinity resistance and tolerance, including genomics, transcriptomics, proteomics, metabolomics and phenomics. Integrating multi-omics data effectively is critical to gaining a more comprehensive and in-depth understanding of the molecular pathways, enzyme activity and interacting networks of genes controlling salinity tolerance in rice. The key data mining strategies within the artificial intelligence to analyse big and complex data sets that will allow more accurate prediction of outcomes and modernise traditional breeding programmes and also expedite precision rice breeding such as genetic engineering and genome editing.

## 1. Introduction

Several countries may face major challenges in achieving food security due to rising populations, pressures on land and water resources, and inadequate infrastructure. Devastating climatic conditions have amplified the biotic and abiotic stresses that aggravate the global crop production challenge [1]. Crop productivity is reduced by abiotic stresses such as salinity, drought, heat and cold [2,3]. Salinisation and soil water deficit are two major stress factors that have a direct impact on crop production [4]. Soil salinity is becoming a global agriculture threat, impeding crop growth, development and yield [5]. Salinity currently affects 6% of land area and more than 20% of farmland. While salinization occurs at about 3 ha/min, this rate increases every year as a result of improper irrigation methods, increased fertiliser use, excessive ploughing and salt intrusion into the coastal zone due to the rise in sea level [6,7]. The effect of salinity varies by genotype, with high salinity causing 30–80% yield losses [8]. Millions of hectares of land in Asia and Africa are ideal for rice production, but they are currently underutilised due to high salt content. Alarmingly, rising salinisation is expected to mean that half (50%) of all arable land will be salinised by 2050, while the population increases concurrently [7,9].

More than half of the world’s population consumes rice (*Oryza sativa* L.) as a staple food [10]. It is grown on approximately 150 million hectares of agricultural land worldwide, yielding nearly 500 million metric tonnes of rice [11]. Due to the growing global population and rice consumption, it is critical to improve rice production. Rice-based cereal is the most salt-sensitive monocot among cereals [12]. Although rice tolerates salinity during the germination, active tillering and maturity stages, it is more sensitive during the early seedling and reproductive stages [13]. The complexities of salinity reactions can cause many changes at the morphological, physiological, biochemical and molecular levels, including osmoregulation, ion homeostasis (mostly Na^+^/K^+^), oxidative homeostasis such as reactive oxygen species (ROS), and effective stomatal function [14].

Significant efforts have been made around the world over the last two decades to better understand the mechanisms of salinity stress and develop salt-tolerant rice cultivars. Understanding the salt tolerance mechanism and the genes involved in the stress signalling network at the whole-plant level is critical for rice improvement. Plant scientists have adopted high-throughput omics platforms (genomics, transcriptomics, proteomics and metabolomics) to study salinity stress at the genetic and molecular levels. Integrated omics techniques have contributed significantly to understanding the effects of salt stress and the adaptations that plants make to survive and mitigate adverse environments [15]. Although several salinity-effective genes in rice have been identified, none of them have been effectively integrated with commercial germplasm because, at the field level, those genes respond differently than in controlled conditions, where multiple factors and stresses are present simultaneously [16].

Multi-omics reveal molecular phenotypes by providing insights into the mechanisms controlling biological processes, molecular functions, interactions and cellular destiny, whether it be in vivo or in vitro. Subsequently, genomic prediction, machine learning, genetic engineering and genome editing all provide novel ways to accelerate more precise pre- and breeding efforts aimed at improving crop resilience and production while meeting future global food demand in the face of rising abiotic stress [17]. Plant biologists have now joined the large-data age due to fast developments in high-throughput genomic data generating technology [18]. Machine learning, which is progressively gaining popularity in biology, provides potential computational and analytical solutions for the integrated study of gene expression levels, proteins, metabolites as well as advancements in modelling techniques to predict agronomically relevant traits under environmental stress conditions [19,20].

Thus, the ultimate aim of reviewing multi-omics applications is to thoroughly understand the salt stress effects on rice and mechanisms behind rice salt tolerance and salt stress signalling. The current review focuses on recent advances in the understanding of the molecular mechanisms of salinity in rice, their effects on rice growth, development and yield, as well as previous omics efforts in understanding and improving salinity traits in rice. We also focus on the integrative application of multi-omics approaches and the role of bioinformatics that can be used to facilitate precision rice breeding, such as genetic engineering and genome editing.

## 2. Effects of Salinity on Rice

Soil salinity is one of the most damaging abiotic stresses that has a direct impact on crop production worldwide, particularly in South Asian coastal regions. The accumulation of excessive salt content in soil impedes rice crop growth and results in plant death. Saline soil is defined as having an electrical conductivity of 4 dSm^−1^ or higher [21] and osmotic pressure of around 0.2 MPa [12]. While sodium (Na^+^) and chloride (Cl^−^) are the dominant ions, saline soil also contains Ca^2+^, Mg^2+^, Na^+^, SO_4_^2−^, Cl^−^, HCO_3_^−^, and a tremendous amount of K^+^, CO_3_^2−^ and NO_3_^−^ soluble salts. The pH of saline soil typically ranges from 7 to 8.5 [22]. The primary causes of salt stress are high concentrations of Na^+^ and Cl^−^ ions in the soil solution. Initial growth reduction occurs as a result of altered water status and salinity-induced ionic and osmotic stress, both of which contribute to growth reduction [23].

**Figure 1:**
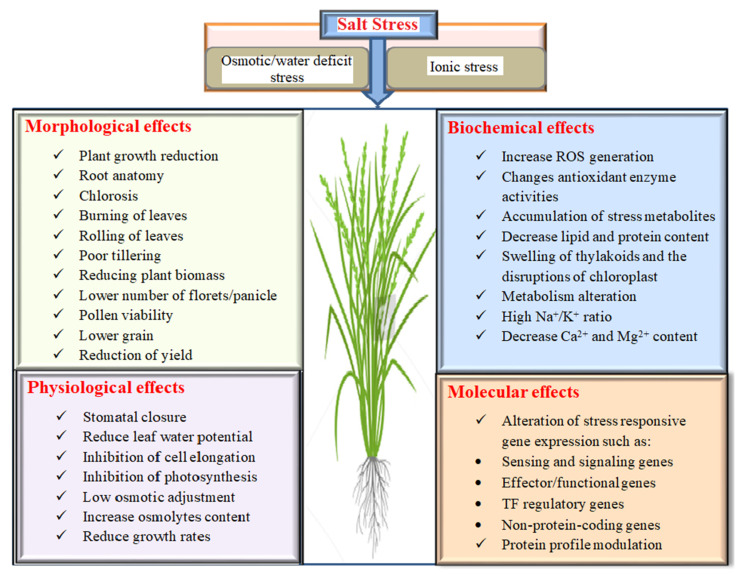
Various salinity effects on rice. At the morphological stage, it causes chlorosis, burning of leaves, rolling of leaves and poor tillering, disturbing plant development. At the physiological and biochemical levels, salinity interferes with critical plant functions such as photosynthesis, respiration and nutritional acquisition, as well as triggers the formation of ROS, which disrupts enzyme activity and impairs membrane integrity. Besides these effects, salinity alters several genes and protein expression profiles related to overall growth at the molecular level.

Except for a few halophytes, most crops, including rice, had lower yields as a result of salt stress. Rice is a popular cereal crop with high economic value, but it has been shown to have the most genetic diversity for salt tolerance due to gene effects [24]. It is classified as the most sensitive monocot to salinity, mainly during the initial seedling and advanced reproductive stages, and severely impacts rice yield via the reduction of major morphological changes [25]. The following headings are considered to have negative effects on rice due to salinity stress. Figure 1 shows the outline of the morphological, physiological, biochemical and molecular effects of salinity on rice.

### 2.1. Morphological Effects on Rice under Salinity

Plants have a wide range of responses to salinity stress. Salinity has a significant impact on the morpho-physiological traits of rice plants [26,27]. Under saline conditions, major effects on rice seed germination, root anatomy, chlorosis, leaf burning, poor tillering, leaf rolling, reducing plant biomass, lower number of florets/panicle, pollen viability and lower grain weight result in significant yield loss [28,29]. Plant height and biomass are reduced by high salt concentrations in rice seedlings due to osmotic, ionic and oxidative stresses [30,31].

Salinity has a significant impact on rice growth, which is also highly dependent on species and growth stage. Rice grain yield is more sensitive to salinity than in the later stages of vegetative advance, even though young seedlings are mostly sensitive to salinity [32,33]. The rice growth phase is highly complex under a salt stress environment because it involves many metabolic changes that may harm grain development.

The flowering stage is critical for determining grain yield and is also a highly growth-sensitive stage in the life cycle of the crop plant. Grain sterility is considered a serious problem in rice grain yield under salt stress conditions [32] and this sterility has been attributed to nutritional deficiencies [34]. Several studies have revealed that salinity stress during fertilisation can cause panicle sterility, which begins to deteriorate in grain settings, decreasing the stigmatic surface, pollen compartment capacity, or both [35]. Pollen viability and carbohydrate content were significantly reduced due to an increase in Na^+^ ions in the rice floral parts. At the time of grain filling, leaf water potential also plays an important role in assimilating the production and partitioning. Grain yield decreases in a salt-stressed environment due to a lack of carbohydrates, resulting in vegetative and spikelet development. However, lower rice grain production under salt stress is caused by a significant decrease in soluble sugar translocation to superior and inferior spikelets, as well as inhibition of starch synthesis during grain development [35]. Rice productivity is reduced by salinity stress in general; however, the consequences vary depending on salt level, environmental conditions, plant types, growth and developmental phases.

### 2.2. Physiology, Biochemistry and Molecular Response of Rice under Salinity Stress

Salinity is one of the severe abiotic stresses on crops, causing osmotic, ionic imbalance and oxidative damage. The first effect of salinity on rice is the osmotic effect, which causes a decrease in osmotic potential, followed by the ionic effect, which causes ion toxicity, ultimately triggering oxidative stress and nutrient deficiencies in rice [36]. The initial loss of rice growth caused by salinization is due to a lack of water [37]. Plant water potential and osmotic potential decrease as salinity increases, whereas turgor pressure increases.

Salinity regulates photosynthesis, which is a critical physiological characteristic for plant growth and development. Chlorophyll is the most important component of photosynthesis. Photosynthesis and chlorophyll content is inversely related to the amount of salt stress [38,39]. Soil salinity has a direct impact on photosynthesis during both the vegetative and reproductive stages [38]. High salt stress lowers the effective PSII quantum yield and results in a lower K^+^/Na^+^ ratio in the cytosol [40]. Chlorophyll content, fluctuations in effective PSII quantum yield and membrane permeability are the major indicators for understanding the effect of salt on photosynthetic efficiency [41]. Chloroplasts are critical organelles that participate in photosynthesis and are more sensitive to salinity [42]. Salt stress ionic and osmotic effects cause and induce swelling of thylakoids and disruptions of the chloroplast envelope in rice, respectively [43]. Ionic stress, on the other hand, has the greatest impact on rice, and can even cause plant death in extreme situations.

The high levels of Na^+^ and Cl^–^ ions in rice plants causes an ionic imbalance and reduces the uptake of other essential nutrients such as K^+^, Ca^2+^ and Mn^2+^ in the cells and tissues [44,45]. Excess Na^+^ competes with K^+^ transport across the plant cell plasma membrane, which is crucial for the catalytic activity of several central enzymes and also essential for osmoregulation, protein synthesis, cell turgor maintenance and adequate photosynthetic activity [46]. No Na^+^ specific sensors/receptors have been found in plants. However, the Salt Overly Sensitive (SOS) signalling pathway is a Ca^2+^-dependent protein kinase pathway and the calcineurin B-like (CBL)/CBL-interacting kinase (CIPK) route has been thoroughly described in model plants of Arabidopsis. In rice, *OsSOS1*, *OsSOS2/OsCIPK24* and *OsSOS3/OsCBL4* have been investigated. *OsCIPK24* and *OsCBL4* work together to trigger the *OsSOS1* gene [47] which excludes Na^+^ from shoots and increases salt tolerance. It was also found out that the majority of rice *CBL* and *CIPK* genes exhibit transcriptional responses to abiotic stressors including such salinity [48]. Of 29 calcium-dependent protein kinases (*CDPK*) genes reported in the rice genome, some of them positively regulate salt, drought and cold tolerance in rice [49]. *OsCPK4* [50], *OsCPK12* [51], and *MDAR* and *DHAR* genes [52,53] enhance tolerance to salinity by reducing the accumulation of ROS. Excessive Na^+^ and Cl^–^ ions uptake in plant cells causes major physiological disorders such as membrane disruption, inability to detoxify ROS, and a decrease in the rate of photosynthesis and antioxidant enzyme reactions [54]. ROS normally acts as a signalling molecule and a by-product of hyperosmotic and ionic stress that induces membrane dysfunction and cell death under both biotic and abiotic stress conditions, and it is also one of the primary causes of cell damage [55]. ROS is a partially degraded form of atmospheric oxygen produced during key processes such as photosynthesis, respiration and photorespiration [56]. A low level of ROS can act as a signal to trigger salt stress responses, whereas excessive ROS build-up causes phytotoxic responses such as DNA mutation, protein breakdown, and starch and lipid peroxidation [57].

To mitigate damage and repair initiated by ROS, plant cells detoxify ROS by upregulating an antioxidative system consisting of enzymatic and non-enzymatic components [58]. Among them, peroxidase (POX), ascorbate peroxidase (APX), superoxide dismutase (SOD), catalase (CAT) and glutathione reductase (GR) are the enzymatic antioxidants while the non-enzymatic antioxidants include water soluble components such as ascorbic acid, flavonoids, glutathione and the lipid soluble components such as α-tocopherol and carotenoids. *OsAPX2* and *OsAPX8* enhance APX activity, lower the H_2_O_2_ and MDA levels, reduce oxidative damage and improve rice salt tolerance [59,60]. *OsGR2* and *OsGR3* increase GSH levels and improve abiotic stress tolerance including salinity [61,62].

Transcriptional control is also an important aspect of plant response to abiotic stresses. In an attempt to increase rice salinity tolerance, numerous transcription factors were examined. Major TF families regulate salt tolerance in rice such as dehydration-responsive element binding protein (DREB), ABA-responsive element binding protein/factor (AREB/ABF) and NAC [63].

On the other hand, Na^+^ entry causes chlorosis and necrosis along with premature senescence in mature leaves through disruption of protein synthesis and intrusive enzyme function [12]. Excess Cl^−^ is toxic to rice plants and reduces grain yield [64], whereas Na^+^ is the primary cause of ion-specific damage in many plants, including rice [65]. Under salt stress conditions, water and nitrogen relationships may influence essential physiological and biochemical changes as well as grain yield in rice. Older rice leaves may accumulate toxic levels of Na^+^ and Cl^−^ ions, influencing photorespiration and reducing NH_4_^+^ production during photorespiration. This may also change the NH_4_^+^ assimilation pathway under salt stress [66]. As a result, salinity disrupts ionic homeostasis, increases ionic toxicity and causes a nutritional imbalance in plants by increasing Na^+^ and Cl^−^ intake, ultimately limiting rice plant growth and development. Plants develop a variety of adaptation mechanisms to counteract the negative effects of salinity, including osmotic adjustment, ion transport and compartmentalization and ion sequestration [67]. These are eventually controlled at the protein level.

## 3. Adaptive Mechanisms of Salinity Tolerance in Rice

Rice is a highly complex plant with many genes involved in its salt response. Since salt tolerance in rice has very complex genetic and physiological characteristics, it is difficult to fully comprehend how it reacts to salt. The mechanism of salinity tolerance in rice can be classified into three categories [12]. The first mechanism is osmotic stress tolerance, which is regulated by long-range signals that reduce shoot growth and stomatal conductance while also incorporating biosynthesis and storage of compatible solutes to maintain water absorption [68]. The second mechanism is ion exclusion, which generally involves Na^+^ and Cl^−^ transport in roots, with Na^+^ transporters reducing toxic Na^+^ accumulation within roots and leaves. This method regulates the loading of Na^+^ into the xylem and the retrieval of Na^+^ from the xylem before it reaches the photosynthetic tissues in the shoot. The third mechanism is tissue tolerance, which occurs when leaves have high salt concentrations but the Na^+^ ions are compartmentalised or sequestered at the cellular and intracellular levels, particularly in the vacuole. This reduces the harmful effect of Na^+^ in the cytosol, the synthesis of suitable solutes and the production of enzymes that catalyse ROS detoxification. In most cases, the plant’s salt stress tolerance relies on all three mechanisms working together rather than just one [12,68,69]. At moderate salinity levels, ion exclusion may be the primary tolerance mechanism of plants. However, ion exclusion may be more effective at higher salinity levels, where tissue tolerance becomes the primary tolerance mechanism.

**Figure 2:**
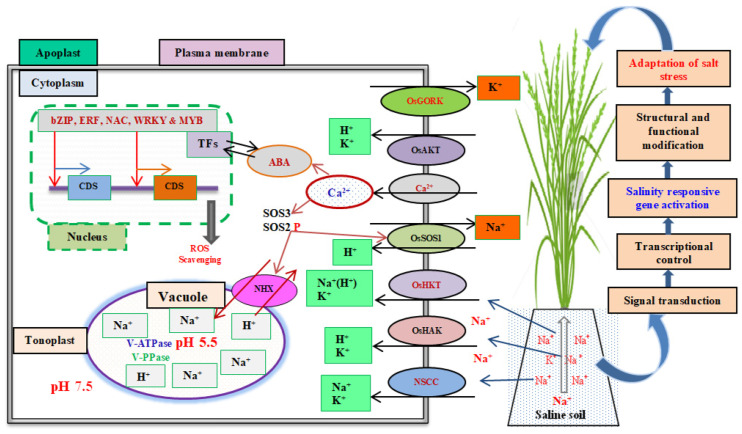
A summary of the ion transport system and adaptive mechanisms of rice under salinity. A schematic diagram of the ion transport system involved in cellular sodium uptake and accumulation in plants; SOS: Na^+^/H^+^ antiporter, HAK: K^+^ transporter, AKT1: K^+^ transporter, HKT: K^+^/Na^+^-symporter or Na^+^ transporter or high-affinity K^+^ transporter, NHX: Na^+^/H^+^ exchanger, NSCC: non-selective cation channel. The sources of energy during salinity stress are vascular proton-pumping pyrophosphatase (H^+^-PPase or V-PPase), vacuolar H^+^-ATPase or V-ATPase: V-type. Ca^2+^-dependent signalling network involves salinity stress response; ABA acts as a major signalling molecule in stress responses.

A summary of the ion transport system and adaptive salinity mechanism of rice is shown in Figure 2. Osmotic concentration and ion-specific stresses are two stresses on plant tissues under salinity conditions. Osmotic concentration stress is higher in the soil than in plant cells, resulting in a water deficit, whereas ion-specific stress caused by altered K^+^/Na^+^ ratios and Na^+^ and Cl^–^ concentrations is harmful to rice. Na^+^ ions enter the plant root channel with water via both symplastic and apoplastic pathways, which are mediated by various ion channels/transporters. Several classes of cation channels have been proposed to mediate substantial Na^+^ entry into plant roots, including outward- and inward-rectifying K^+^ selective channels, non-selective cation channels and high-affinity potassium transporters. The stealth of Na^+^ entry is due to the physiochemically similar monovalent cations, which make distinguishing between the two ions of *OsHKT* transport proteins difficult. As a result, the plant responds to the maintenance of low cytosolic Na^+^ concentrations and a high cytosolic K^+^/Na^+^ concentration ratio by *OsHKT*, *OsHAK* and *OsAKT1* transport proteins [12,70].

Increases in the level of Na^+^ in the cytosol to maintain ion homeostasis in plants mainly relies on signalling pathways. The Salt Overly Sensitive (SOS) pathway is a Ca^2+^-dependent protein kinase pathway that regulates ion homeostasis via Na^+^/H^+^ antiporters SOS1, SOS2, SOS3 and SCaBP8. Excess Na^+^ ions are exported under salinity conditions as a result of cytosolic Ca^2+^ signals. The Ca^2+^ binding proteins SOS3 and SCaBP8 decode the Ca^2+^ signals and translate them directly to a serine/threonine-protein kinase, SOS2. The SOS3 and SCaBP8, which are expressed in the roots and shoots, respectively, interact with and activate SOS2 in the plasma membrane. This SOS2 then phosphorylates and activates SOS1, increasing Na^+^/H^+^ exchange activity and thus salt tolerance [29,71]. SOS1 is important for the transport Na^+^ to apoplasts from the cytoplasm, and the SOS2-SOS3 complex regulates SOS1 expression. Under salt stress conditions, the SOS3-SOS2 complex also positively regulates NHX, a vacuolar Na^+^/H^+^ exchanger that transports excess Na^+^ from cytoplasm to vacuoles via vacuolar H^+^ pump-ATPase and H^+^-pyrophosphatase (PPase) [67,72,73]. Other signalling compounds, including nitric oxide, hydrogen sulphide, hydrogen peroxide, ROS and growth regulators such as abscisic acid (ABA), ethylene, jasmonic acid and salicylic acid, play important roles in cell signalling tolerance to not only salinity but also multiple stresses [74].

ABA is a phytohormone that functions as a central regulator in ion homeostasis. High salinity stimulates ABA biosynthesis. ABA-dependent regulation of salt-responsive genes and ABA-responsive TF that binds to core cis-acting elements are also important in rice salinity stress. The ABA-responsive independent ROS scavenging system is involved in salinity tolerance in rice because ROS accumulation disrupts ion homeostasis and causes oxidative damage in the cells [29,75,76]. SOS and ABA-responsive pathways are involved in salt tolerance but their precise regulatory mechanisms are unknown. Since salinity tolerance in rice is polygenic, there is a greater need to use high-throughput biotechnological tools to characterise Na^+^-specific sensors/receptors, novel transporters and channels, and salt-related genes’ screening.

## 4. Omics Platforms Used for Rice Improvement

One of the primary issues in using modern approaches to ensure nutritional food security is the development of rice cultivars for salinity resistance and tolerance. Modern omics platforms in plant biology have gained traction over the last two decades in studying molecular mechanisms at the cellular, tissue-specific or organism-level to gain biological insights in response to stress [77]. A relatively new field of life science known as system biology is being used to address the integration of diverse omics, providing multidimensional biological information. Rice genome sequences, high throughput technologies, computational tools and omics enable plant biologists to fully understand and uncover regulatory mechanisms that can be used in rice research for gene mining and breeding to develop plant functions. Recent advancements in rice salinity tolerance have been made possible by the use of the omics tools listed in Table 1 and Table 2.

### 4.1. Genomics

Genomics is the systematic and comprehensive study of DNA on an organismal scale that provides a framework for mapping, nucleotide sequence analysis, genome structure and composition, and genetic variation studies [78,79] to improve breeding efficiency and genetic improvement [80].

Structural genomics includes sequence polymorphisms and chromosomal structure, which allow the creation of a physical and genetic map to reveal the trait of interest based on molecular markers. The rice whole genome sequence assists researchers in functional genomics by providing insights into gene activities related to the control of the trait of interest in response to salt tolerance in rice [81]. Salinity is a highly complex physiological trait that is genetically controlled by QTLs [82]. QTLs for salinity tolerance have been identified in rice cultivars using amplified fragment length polymorphisms, restriction fragment length polymorphisms, simple sequence repeats (SSRs) or microsatellites, and single nucleotide polymorphism (SNP) markers [82,83]. Several QTLs in rice have been identified and linked to salinity tolerance. A few important QTL mappings have been completed using a genomic approach. Salt tolerance QTLs are mainly identified at the seedling stage and mature stages, with few reports on the germination stage in rice (Table 1).

**Table 1:** Rice QTLs linked to salt tolerance.

Parents	QTLs Number	Different Stage	References
Pokkali × IR29	23	Seedlings	[84]
Nonabokra × Koshihikari	11	Seedlings	[85]
Ahlemi Tarom × Neda	73	Seedlings	[86]
Capsule × BRRI dhan29	27	Seedlings	[87]
Pokkali × Bengal	50	Seedlings	[82]
Hasawi × IR29	34	Seedlings	[88]
Kalarata × Azucena	13	Seedlings	[89]
Nonabokra × Jupiter	33	Seedlings	[90]
Dianjingyou × Sea Rice 86	1	Seedlings	[91]
CSR27 × MI48	25	Seedlings, vegetative and reproductive	[92]
OM7347 × OM5629	9	Seedlings, vegetative and reproductive	[93]
Horkuch × IR29	14	Seedlings and reproductive	[94]
Cheriviruppu8 × Pusa Bashmati 1	16	Reproductive	[95]
Pokkali × IR36	6	Maturity	[96]
CSR27 × MI48	8	Maturity	[97]
Jiucaiqing × IR26	16	Germination	[98]
Wujiaozhan × Nipponbare	13	Germination	[99]

Three leading Saltol QTLs flanked by RM140 and C1733S were identified on chromosome 1 from a cross between Pokkali and IR29 indica rice varieties for ion absorption, Na/K ratio and their significant contribution to salt tolerance [100]. These three common QTLs accounted for 39.2%, 43.9% and 43.2% of total phenotypic variation. The two most robust QTLs, qSKC-1 for shoot K^+^ concentration and qSNC-7 for shoot Na^+^ concentration, were discovered by crossing indica (Nonabokra) and japonica (Koshihikari) varieties, and they accounted for 40.1% and 48.5% of total phenotypic variance, respectively [85]. The *SKC1* gene was isolated using map-based cloning and found to be a member of the HKT transporters in rice, which is involved in regulating K+ homeostasis under salinity stress conditions [96]. A QTL (qST1.1) was recently identified on chromosome 1 that significantly contributes to salt tolerance in indica “Sea Rice 86” with 62.6% of phenotypic variation [91]. This QTL will not only help to understand salinity mechanisms but will also help accelerate future breeding practices. Mapping, cloning and QTL identification are among the next steps in associating salinity tolerance in rice [13].

Investigating the genetic architecture of species and population divergence aids in understanding how lineages develop and adapt, and hence how recurrent evolutionary forces are [101]. Recent sequencing techniques have shown that the genomic repercussions of divergence are mixed and, in some cases, deceptive [102]. Genomic-assisted characterization such as Genome-Wide Association Studies (GWAS), Genome Environment Associations (GEA) and Genome-Wide Selection (GWS) has consistently aided in exploration of adaptation to climate change and enhancing further Marker-Assisted Selection (MAS) for abiotic stress tolerance crop improvement [103,104]. GWAS is a powerful technique that might reveal variants linked to traits. Based on SNPs in the sequencing data, GWAS studies may potentially discover correlations between genetic variants/phenotypes in any organism’s population [105]. There are various GWAS applications in rice with unique growth stages and features in saline environments. Recently, GWAS application found 23 Marker–Trait Associations in rice salinity tolerance at early vegetative stage [106]. Using GWAS, 20 QTN were found, among 6 and 14 associated with salt tolerance at germination and seedling stage, respectively [107]. High-density SNPs were utilized over the last few years to find variants with the GWAS approach for rice improvements. Although many traits related to abiotic stress are controlled by many polygenes, those are undetectable in single-locus GWAS models. Later on, muti-locus GWAS methods were used to identify salt tolerant loci in rice at seed germination stage. A total of 371 QTNs were identified related to salt tolerance. Furthermore, based on functional annotation, 66 genes were detected in the proximity of the 56 QTNs [108]. Therefore, the multi-locus GWAS is very useful for the detection of salt tolerance loci in rice.

Several biotechnological approaches have been developed to isolate novel salinity-related candidate genes, characterise the genes and perform functional analysis via overexpression [109]. Several rice genes have been functionally identified by genetic analysis [110]. Massive online plant genomic data databases, libraries and archives serve as a basis for transcriptomics, genome engineering and proteogenomics [111]. It is possible to improve forecast accuracy and accelerate genetic improvements by reducing the breeding cycle and combining high-performance phenotyping with genomic data. In this regard, genomics is a valuable tool for deciphering both evolutionary and functional characteristics of genes of interest by using rice genome sequences. Next-generation sequencing (NGS) has accelerated rice genomic research by identifying and utilising QTLs and candidate genes that regulate agronomic traits. Unlike traditional breeding techniques that can take years to produce a new cultivar, NGS allows for effective genetic mapping and genome analysis [112].

Metagenomic and epigenomic tools have been developed as new omics branches to improve growth and grain yield in response to environmental stresses [80]. Metagenomics is a new omics branch that investigates mutational processes that coordinate genetic change in mutant traits. SAGE (serial analysis of gene expression), HRM (high-resolution melt), TILLING (target induced local lesions IN genomics), and microarray can be used to study such mutational events [113]. Microarray analysis has proven that mutagenesis is an important approach for identifying gene functions and developing a wide range of desired agronomic characteristics [114]. Mutation breeding contributes significantly to the development of climate-resilient salinity-tolerant varieties with high yields [115]. As a result, it can be used as an important tool in rice functional analysis and the creation of genetic variability to improve traits [116]. Biological, physical, and chemical agents are used in crop mutagenesis [117]. Several successful applications have been made to improve salt tolerance. A set of mutagenised lines can be derived from chemical mutagens such as EMS-induced rice mutants [118]. The new mutant line named salt hypersensitive 1 (shs1) was developed after being treated with sodium azide, and it plays an active role in cellular Na^+^ ion homeostasis and antioxidant mechanisms [119]. Genome duplication increases root tolerance to salinity stress by improving proton transport, which may aid in reducing Na^+^ entry into the roots [120]. Nakhoda et al. used chemical mutagens to develop rice mutants; tolerant mutants have lower Na^+^ and higher K^+^ absorption capacities, indicating a higher K^+^/Na^+^ ratio in their shoots than sensitive mutants [121]. Physical agents, such as ionising radiation, are used more frequently in rice research than chemical agents; these techniques generate ROS that interact with DNA, resulting in oxidative damage, nucleotide changes and single and/or double-strand breaks [122]. Two rice mutants, ST87 and ST301, were produced as a result of gamma irradiation; the physiological characterisations of these mutants revealed that they are more salinity tolerant than the wild type [123]. Joshi et al. found that gamma-irradiated rice mutants produce more biomass and increase yields under saline conditions [124]. These mutants will be the most useful for future research into the novel genes that regulate biomass and yield traits under saline conditions. Gamma irradiation is a valuable tool for increasing genetic variability, which may result in improved traits without changing crop phenotype [125]. Both functional genomic and metagenomic techniques are highly beneficial in terms of rice growth, yield improvement and salt resistance.

Epigenomics refers to the study of chromatin modification or remodelling patterns across the whole genome. It mainly consists of DNA or small RNA methylation and histone modification at the genome level that can result in inheritable phenotypic variations [126]. DNA methylation has also been identified as a critical component in plant genomic responses under various environmental stimuli [127]. Plant DNA methylation, histone modification and non-coding RNA are epigenetic mechanisms that regulate chromatin structure and gene expression in response to environmental stimuli [128]. Methylation-sensitive amplified polymorphisms (MSAP) and bisulfite sequencing were used to quantify DNA methylation and identify the methylation status in the rice genome under salt stress [129]. Salinity affects DNA methylation in retrotransposons, chromatin modification and stress-responsive genes scattered on rice chromosomes, as well as cytosine methylation and gene expression. Pokkali, a well-known salt-tolerant rice germplasm, was found to be more capable of changing DNA methylation levels in response to salt stress than the IR29 sensitive variety [127]. The MSAP approach was used to characterise the DNA methylation alterations under saline conditions in introgression lines IL177-103 (salt-tolerant) and IR64 (salt-sensitive), and the results revealed a few locations with permanent DNA methylation changes. Major salt-induced DNA methylation changes persisted even after recovery [130]. A set of differentially methylated regions (DMRs) in salt-tolerant cultivars under salinity stress were recently discovered [131]. DMRs appear to influence gene expression in their immediate proximity. It was also hypothesised that the identified DMRs could regulate chromatin structure and modulate gene functions. Many rice epigenetic regulators have been discovered and shown to be involved in a wide range of cellular growth and stress-response pathways [126]. As a result, epigenomics can have a significant impact on rice improvement in response to salinity stress.

Pangenomics refers to a species’ whole genome composition, which can be divided into core and non-core genes [132]. The core genes are preserved and play a key role in carrying out the critical functions within the species. Non-core genes, on the other hand, provide crop genetic diversity as well as a variety of agronomic characteristics that aid in crop survival in adverse climatic conditions [133]. The comparison of foreign and wild cultivars is aided further by pangenome analysis of non-core genomes, which allows researchers to examine genes in wild species that were lost during crop domestication. This technique can capture unique genes that were not found in the reference genome, potentially leading to increased salt tolerant cultivars to solve food security issues in the context of climate change [132]. There has been no pangenome study to date to identify and map salt tolerance genes in rice. Pangenomic research is urgently needed to mine novel genes in wild relatives to mitigate the salinity problem.

A phenotypic analysis is used in functional genomics. Combining genomics and phenomics aids in obtaining complex trait information to identify numerous QTL for crop improvement [134]. The GWAS technique has been used to identify the controlling QTL complex for rice salinity tolerance [135]. Subsequently, a combination of QTL mapping, GWAS and RNA-seq aid in identifying candidate genes in rice [136]. GWAS with a metabolome has proven to be an effective tool for dissecting a variety of secondary metabolites to adapt to different environmental stresses [137].

### 4.2. Transcriptomics

Transcriptomics is defined as the study of RNA transcripts in cells or tissues in response to various physiological or environmental stimuli [138,139]. Transcriptomics investigates RNA levels across the genome, both qualitatively and quantitatively. Diverse mechanisms regulate gene expression under salinity stress. The technique is useful for researchers to understand differential expression at the transcript level and provides an understanding of gene structure, gene expression regulation and its function, and genome dynamics [140]. RNA-seq and microarrays are two modern, contemporary key techniques for identifying genes that are expressed differentially [141,142]. Microarrays enable the simultaneous analysis of thousands of transcripts that can be counted among a set of predetermined sequences [143]. Microarrays and tag-based sequencing techniques have been used to investigate gene expression patterns in various plants, including rice [144,145,146]. These methods were used to identify a set of known stress-inducible genes, and it was proposed that those genes would be the most useful candidates for transgenic salinity tolerance rice improvement [147]. Using a cDNA microarray, 486 salt-responsive expressed sequence tags were identified in rice shoots under salt stress conditions, with the majority of them being novel, indicating that there are a large number of salt-induced genes [148].

High-throughput next-generation sequencing is a revolutionary tool in transcriptomics that can overcome the limitations of array-based approaches because it can capture all sequences [143,149] and its popularity grew surprisingly after 2008 when more advanced Illumina technologies recorded 10^9^ transcript sequences with accurate quantitation [150]. Later, PacBio and Oxford Nanopore Technologies came to dominate plant genome studies due to their high-quality sequences, large sequence reads and lower error rates [151]. Remarkable stress-inducible transcripts were also identified in rice using RNA-seq. Transcriptome analysis of Dongxiang wild rice leaves and roots under salinity stress compared to non-stress conditions was conducted to unravel stress-tolerance mechanisms [152]. The study found many salt stress-inducible genes that are co-localised on fine-mapped salt-tolerant linked QTLs, opening up the possibility of gene cloning and elucidating the underlying molecular mechanisms in response to salt stress. Another transcriptome analysis of rice seedling roots under salt stress revealed 447 upregulated genes [153]. Metabolite analysis indicated that phenolic and flavonoid content increased in the root during salt stress. Jahan et al. used RNA-seq to analyse transcriptome profiling and heterosis-related genes in mega hybrid rice LYP9 and its two parents in salinity and control levels and found 8292, 8037 and 631 salt-induced DEGs [154]. The findings suggest that hybrids play an important role in responding to salinity stress, providing a new perspective on heterosis mechanisms in salinity tolerance.

Many genes and transcription factors (TFs) that are either upregulated or downregulated in response to salt stress have already been identified using transcriptomics and genomic approaches [155]. Similarly, gene expression has been shown to modify various TFs in rice. The *C2H2* type zinc finger TF was discovered in rice as a novel TF that modulates stomatal aperture for drought and salt response [156]. According to rice research, a variety of transcription factors are implicated in the response to salt stress, such as *OsMYB91*, *OsWRKY42*, *OsbZIP71*, *OsTZF1* and *OsNAC5* [157,158,159,160,161]. A large number of rice genes have been identified and characterised based on ion transport or ion homeostasis, antioxidants, signalling and molecular chaperons that are upregulated in response to salinity [162]. The *OsSOS1*, *OsHKT1;5*, *OsHKT2;1*, *OsNHX1*, *OsAKT1*, *OsNRT1;2*, *OsTPC1*, *OsCDPK7*, *OsARP*, *OsMAPK5*, *44* and *OsSERF1* genes have been identified as being regulated during salt stress in rice [76].

The data from RNA-seq can be used to find genetic SSR markers that aid in marker-assisted breeding to improve agronomic traits under different stress conditions. Transcriptome sequencing analysis of black rice seed tissues was used to develop SSR markers. These markers are beneficial in terms of genetic diversity, QTL mapping and marker-assisted breeding [163]. A study of RNA-seq data of rice under salt, drought and cold SSR stress was conducted, and the result suggested that genes with altered SSRs can be used as functional biomarkers [164].

Comparative transcriptomics is another method for investigating differential expression patterns in response to salt stress. Previously, a comparative analysis was conducted on salt tolerance and sensitive cultivars to better understand the regulatory mechanisms. The results revealed that members of the *C2H2* and *bHLH* TF families have increased expression, suggesting that they may be controlling genes involved in wax and terpenoid metabolic pathways [10]. A comparative leaf transcriptome study on rice seedlings was also conducted to better understand salt stress, and 1375 new genes and 286 differentially expressed genes that are only found in tolerant cultivars were discovered [165]. Cartagena et al. conducted a comparative transcriptome analysis on Mulai (tolerant) and IR29 (sensitive) root types [166]. More transporters, such as ion- and sugar-related transporters were identified in Mulai roots, and they play a role in the regulation of salt tolerance.

In reality, the mRNA level of data only indicates how gene expression is regulated in the cell, and it must be mutually reliant on the proteomic level of data, which is frequently more useful in determining biological functions because plant stress responses are mediated by proteins. Rice has recently been subjected to comparative transcriptomics and proteomics analysis [167]. It was suggested that comparative analysis aids in the discovery of new salt-responsive genes and unravels gene regulatory mechanisms at the molecular level. Similarly, combining transcriptomics and proteomics can reveal how stress response elements mediate transcriptional and translational levels. The integration of multi-omics platforms (transcriptomics, proteomics and/or metabolomics) in rice was used to identify genes, proteins and metabolites [168]. The findings revealed that the integrated approach aids in understanding cellular responses to stress.

### 4.3. Proteomics

Proteomics is the systematic evaluation or provision of a platform for the global investigation of total expressed proteins by a specific cell, tissue or organism over a specific period [169]. Proteomics is more accurate and comprehensive than genomics and transcriptomics for identifying and quantifying proteins in a specific biological state, as well as assessing post-translational modification, cellular origin and mode of action [170].

Since the initial rice proteome research in the 1990s, significant progress in protein isolation and characterisation has been achieved. However, proteomics is still limited to the cell or tissue parts because protein structure and expression are constantly changing as a result of time, location and response to stimuli. More advanced high-throughput proteomics technologies, such as protein microarrays, gel-based approaches, mass spectrometry, X-ray crystallography and NMR spectroscopy, have already been identified. The most widely used technologies in current proteomics studies are mass spectrometry with LC-MS-MS and MALDI-TOF to identify differentially expressed proteins and protein quantification in response to abiotic stress and stress-responsive pathways [169].

Proteomics has emerged as a powerful tool for molecular phenotypic characterisation, discovering novel genes, the significance of PTMs and interactions and the understanding of the relationship between genotype and functionality [171]. This information further accelerates the breeding programme by identifying precise prospective biomarkers that are used to isolate candidate genes to be incorporated via proteomics-based marker-assisted breeding and gene pyramiding. The re-annotation of the rice genome was aided by well-known proteomes, which revealed novel protein functions. The latest advances in proteomics aid in the discovery of more regulatory proteins and contribute to the development of stress-tolerant rice.

Many researchers investigated proteomics patterns in different rice tissues under salt stress, including leaf sheath, root, leaf, stem, anther, young panicle and various germplasms [172,173,174,175,176,177,178,179,180]. Some proteins are expressed differentially in rice roots and leaf parts after salt treatment, and these proteins may act as salt-stress resistant [158]. Six novel salt-responsive apoplastic proteins were identified using systemic proteomic approaches. Among them, *OsRMC* abundance increases rapidly during the early stages of salt stress. It has been suggested that plant apoplastic proteins may have an essential function in salt-stress signalling [181]. Li et al. used 2-DE and MALDI-TOF MS techniques to conduct proteomics analysis on rice in response to high salt stress [182]. They discovered that 57 responsive proteins were regulated during salt stress, including several novel salt-responsive proteins. Liu et al. used the proteomic approach and classical biochemical methods to analyse the salt response in two rice varieties [183]. They discovered that proteins are expressed differently in tolerant and sensitive cultivars. The findings also indicate that two proteins involved in salt stress response and the ubiquitin 26S proteasome system may improve salt tolerance. According to root-specific proteomics analysis, ubiquitination of proteins alters the protective mechanisms in rice seedlings to withstand salt stress during the early phase [184]. The use of phytohormones such as gibberellic acid (GA_3_) and ABA rice improves salt tolerance. This proteomics analysis was conducted using 2D PAGE and MALDI-TOF MS [185]. Eleven differentially expressed proteins were identified, including enolase, glutamyl-tRNA reductase, salt protein, chaperonin 21 precursor, isoflavone reductase-like protein, ribulose bisphosphate carboxylase and phosphoglucomutase. Some of these proteins are involved in metabolic pathways such as photosynthesis and glycolysis; others, particularly those involved in rice salt response, were discovered to be novel proteins. Sedoheptulose-1,7 bisphosphate regulates the photosynthetic Calvin cycle in rice roots and is generally downregulated in response to abiotic stress and upregulated in response to cadmium [186]. These findings suggest that metabolic pathway modulation is a common strategy for plant abiotic stress tolerance. Under salt stress conditions, 40 protein spots were upregulated in ABA-treated rice seedlings [187]. Most proteins were uniquely upregulated and involved in energy metabolisms, defence and primary metabolisms, and so on. The identified proteins may also lead to improving salt tolerance in rice. Fourteen proteins involved in rice seed inhibition under salt stress that are related to storage and energy supply were identified using a proteomic analysis [188]. The identified proteins can be used to improve seed germination in rice under salt stress. Xu et al. identified 56 differentially expressed proteins in rice shoots under salt stress using quantitative proteomics analysis [189]. Sixteen of them were discovered to be involved in antioxidant, photosynthesis and oxidative phosphorylation pathways. These studies contribute to a better understanding of rice photosynthesis and PSI functions in response to salt stress.

The rice cyclophilin (*OsCYP2*) gene improves salt tolerance in transgenic seedlings when overexpressed in comparative proteomics studies [190]. Some of the proteins can improve plant salinity tolerance. Salt stress in rice may cause a significant increase in fructose 2,6-bisphosphatase (F26BPas) [191]. Plasma-membrane-linked proteins are essential in maintaining intracellular ion homeostasis and plant adaptation to salt stress [67]. The salt-responsive proteins and biochemical properties of two different rice genotypes were investigated using iTRAQ-based protein profiling [192]. Under different salt conditions, 5340 proteins were found in both genotypes. Functional characterization suggests that differentially expressed proteins are involved in salt stress regulation, oxidation–reduction response, photosynthesis and carbohydrate metabolism. A shotgun proteomic approach was used by Lopez et al. to identify more than 2000 proteins in both the root and shoot of the salt-tolerant elite line FL478 during an early salinity stage [193]. Some of the identified proteins are potential candidates involved in the amino acid synthesis, antioxidant stress, mitochondrial activity maintenance, metabolism and the Calvin cycle. To investigate the role of the hpa1 mutant in salt resistance at the molecular level, Xiong et al. used iTRAQ-based comparative protein profiling to identify differentially expressed proteins between the hpa1 mutant and its wild type under salinity stress [194]. There were 4598 proteins discovered, with 279 of them being up- and downregulated. Further functional analysis suggested that 279 proteins are involved in oxidative phosphorylation, photosynthesis, phenylpropanoid biosynthesis, post-translational modification and energy metabolism. Combined proteomics analysis has been used to identify the proteins and salt responsive network in rice. An integrated study of existing proteomics findings from 34 different plant species, including model plant rice, identified about 2171 down- and upregulated protein identities encoding 561 unique proteins in response to salt stress [27]. These newly discovered proteins provide more information about the complex cellular and molecular mechanisms that underpin salt stress response or tolerance. Liu et al. identified 106 and 521 proteins using DIGE- and iTRAQ-based proteomics techniques, respectively [195]. Further metabolomics analysis revealed salt-induced and developmental changes in rice suspension culture cells at the metabolite level. Integrating proteomics and metabolomics approaches will improve our understanding of complex salt-response networks, allowing researchers to identify novel proteins and metabolites for durable tolerant rice.

### 4.4. Metabolomics

Metabolomics is the study of a full set of small molecules or metabolites, which are related to the measurement of biological compounds synthesised or degraded in organisms [196,197]. The data were combined using a robust next-generation sequencing approach and metabolite quantification to develop crop improvement strategies [198]. Metabolites are by-products of cellular reactions that reflect the biological system’s responses to environmental changes [199,200].

Proteomics only detects gene products, whereas metabolomics can evaluate protein expression metabolically and uncover biochemical mechanisms that are important for gene function [201]. Metabolomics results should be combined with transcriptomics and proteomics in a single pipeline to understand the entire plant system [202]. It is useful in studying stress biochemistry in plants and other organisms by detecting various compounds, stress-responsive metabolites and stress signal transduction molecules in plants [190,203,204]. Several modern and high-throughput metabolic fingerprinting techniques were conducted to quantify metabolites in plants, including nuclear magnetic resonance (NMR) [205,206], gas chromatography–mass spectrometry [207,208], liquid/gas chromatography–MS (LC/GC-MS) [209], capillary electrophoresis–MS (CE-MS) [209], ultra-high-resolution Fourier transform ion cyclotron MS [210] and Fourier transform–IR (FT-IR) [211]. GC/LC-MS techniques are the most widely used in plant metabolomics research due to their suitability and sensitivity [212,213].

Secondary metabolites are highly useful in response to environmental stress [80]. Dimethylsulfonium molecules, sugar, amino acids, polyols mannitol and sorbitols are both biotic and abiotic metabolites that act as osmolytes and have the antioxidant capacity to protect plants from severe salinity drought and desiccation conditions [203]. Rice roots responded quickly to salt stress by changing a wide range of energy metabolisms while also inhibiting GA signalling, which may be responsible for rapid root growth capture and development [202]. Ion transport and metabolic components of rice performance are also connected with soil salinity [214].

Rice metabolomics studies identify the types and quality of metabolites that promote seed germination, metabolite variation, metabolic profiling at different development stages and natural metabolite dissimilarities among different rice cultivars [215,216,217,218]. Several studies on salt-affected roots and leaves from 18 genotypes of rice metabolic profiling revealed that salt affects the xylem sap metabolome, significantly reducing the amount of tricarboxylic acid cycle intermediates, organic acids and the shikimate pathway [219]. Nam et al. identified five salt-sensitive metabolic markers in rice roots using H-NMR spectroscopy [220]. Salt stress altered several metabolite accumulations such as glutamate, proline, valine, aspartate, lactate, malate and others that play a critical role in salt tolerance [221]. Metabolite accumulation is differently regulated, indicating a dynamic and differential metabolic response to salinity stress [222].

Another study found that as salt stress increases, so does the amount of reducing sugar and proline, whereas non-reducing sugar, chlorophyll and grain production decrease [223]. Serotonin and gentisic acid are two significant biomarker molecules generated in NaCl-tolerant cultivars [224]. Xie et al. used the GC-MS approach to investigate the molecular mechanisms underlying salt tolerance. In total, 84 metabolites from rice leaf were identified in both saline and normal conditions, including amino acids, organic acids, sugars and small molecular elements [225].

Under control conditions, more amino acids were enriched in tolerant lines than in sensitive lines, implying that tolerant and sensitive lines have different basal metabolite levels. Significantly higher allantoin levels were found in tolerant lines under both conditions, indicating that allantoin is necessary for rice growth. Similarly, levels of sorbitol, pipecolic acid and melezitose increased significantly under salt stress conditions in five rice lines, indicating that they play a key role in salt stress response.

Metabolites have a wider range of chemical structures, properties and functions than DNA, RNA and protein, which are structurally and functionally quite homogeneous. The accumulation or non-build-up of a particular metabolite is responsible for the tolerance and vulnerability to abiotic stress in rice.

A combined transcriptomic and metabolomic approach has made significant progress in revealing the molecular mechanisms underlying improved salt tolerance in rice. Wang et al. compared the transcriptome and metabolome profiles of two rice genotypes grown in salt and salt with ABA [226]. Salt specifically upregulated genes involved in several salt tolerance pathways, including cytoplasmic transport, vacuole sequestration, ABA-mediated cellular lipids and fatty acid metabolic activities, detoxification with cell-wall remodelling in shoots, and oxidative reduction in roots. Xie et al. discovered that integrated exogenous melatonin improves rice salt tolerance by activating phytohormone signalling and specific transcriptional cascades, which work in tandem with numerous antioxidants and distinct metabolic pathways [227]. The metabolic profiling of rice under salt stress conditions was studied using combined transcriptomics and metabolomics data [228]. The results suggest that multi-omics analysis is an effective method for understanding rice metabolic responses to salt stress. Comparative transcriptome and metabolome profiling revealed the molecular pathways underlying *OsDRAP1* in response to salt stress [229]. Several genes involved in transcriptional control, organelle expression and ion transport were significantly upregulated in response to salt stress, as was the number of metabolites such as amino acids, organic acids and various secondary metabolites accumulated in *OsDRAP1* over the expressed line, indicating that they play an important role in salt tolerance. The combination of transcriptomics and metabolomics data can provide more precise information on the molecular pathways driving rice salt tolerance. It has been demonstrated that integrated omics is important in the response of plants to abiotic stress [230,231]. As a result, combining metabolomics with genomics, transcriptomics and proteomics allows for a better understanding of the mechanisms behind the complex architecture of agriculturally important phenotypic characteristics.

### 4.5. Phenomics

Phenomics is the systematic study of phenotypes, which is related to the measurement of physio-biochemical traits of an organism in response to genetic modification or variation and environmental impacts. Phenomics is a novel discipline in plant biology that aids in the collection of high-dimensional phenotyping data at various levels, allowing full characterisation of a genome’s full set of phenotypes with whole genome sequencing [232]. Although the plant phenome can define interactions between genome, environment and management, this phenomenon is also known as genotype, phenotype and environmental interactions [233].

Plants with tolerant traits are valuable genetic resources that can be used to discover alleles via high-throughput sequencing. The two most common approaches for salinity screening are invasive and non-invasive techniques. An invasive technique is commonly used for QTL mapping and the introgression of salt-tolerant genes for rice varietal development [234]. Reliable, automatic, multifunctional, high-throughput non-invasive imaging systems have been recently developed for detecting quantitative and qualitative changes induced by salt stress [235,236,237,238]. It also refers to the accumulation of phenotypic alteration that supports adaptability in classical phenotypic selection [239]. These techniques have enabled rapid assessment of complex traits such as plant height, tiller number and yield, and tolerance to abiotic stresses under both glasshouse and field conditions [239]. Several image-based techniques have been used for phenomic studies such as visible light, hyperspectral, infrared, fluorescence imaging and X-ray tomography [240]. These image-based techniques, when combined with advanced software systems, have emerged as cutting-edge tools for plant biology [241].

Automated imaging techniques are imperative, less time-consuming and efficient for measuring salinity effects [242]. The Red-Green-Blue (RGB) tool, which is based on visible light, is used to evaluate plant canopy or shoot phenotyping and root systems in response to various stresses [243,244]. The combination of IR, RGB and fluorescence systems creates a new platform for the detailed study of rice genotypes in response to salinity [245]. Rice plants respond to salinity in two phases: the osmotic phase and the ionic phase. The effects of osmotic and ionic components can be easily distinguished under salt stress conditions using image-based phenotyping [245]. Several studies have been conducted using non-destructive phenotyping techniques to detect salt toxicity in response to rice. A non-destructive image-based phenotyping technique revealed different effects of salinity under various stress conditions in two rice varieties, indicating that cultivars have different tissue tolerance mechanisms [246]. The image analysis aids in the differentiation of various aspects of salinity, which is a very powerful tool for physiological and genetic studies to elucidate processes that improve rice salt tolerance. Non-destructive imaging technologies enable the identification of new traits and salinity tolerance genes in rice breeding lines by pyramiding for tolerance mechanisms [246]. Siddiqui et al. used infrared imaging to characterise rice phenotypes under salt stress conditions [247]. According to a correlation study of traditional and modern techniques, leaf temperature changes can be a valuable tool for detecting stress-resistant genotypes under salt stress conditions, as well as saving time, being non-destructive and covering a large area. Rice root system architecture (RSA) affects plant growth and survival. Since the root is directly connected to the soil, it must first fight salinity. A non-destructive imaging system was used to identify significant traits for subsequent QTL analysis to understand the genetic mechanisms driving RSA, and the RSA data can be used to investigate genotype–environment interactions [248,249]. Topp et al. used 3D phenotyping and QTL mapping to identify core regions that regulate rice root architecture [250]. Yichie et al. investigated how salinity tolerance differs across accessions of two indigenous Australian wild rice species with *Oryza sativa* cultivars using both destructive and non-destructive-based phenotyping approaches [251]. They stated that non-destructive-based phenotyping is a useful tool for quantifying plant response to abiotic challenges. It was also highlighted that exotic germplasm can provide novel genetic variation for rice salt tolerance. Combining high-throughput phenotyping with GWAS or functional mapping and genome prediction enables the identification of QTLs at both the seedling and reproductive stages, as well as the dissection of the genetic basis of complex multigenic traits in response to rice salinity [235,236,252,253]. Multifunctional and hyperspectral techniques can be used for high throughput phenotyping (HTP) in rice [254]. HTP application with next-generation sensors may lead to improved agricultural productivity, stress tolerance and management in the near future [255]. As a result, phenomic applications combined with other omics may be critical in evaluating phenotypic characteristics in plants under abiotic stress conditions.

**Table 2:** A review of recent omics platforms used in the rice salinity study.

Omic Approach	Techniques	Description	References
Genomics	Map-based sequencing	Rice genome sequence.	[81]
	Illumina-seq	213 and 436 transcript tags of shoot and root were differentially expressed in response to salt.	[256]
	Genome-wide meta-analysis	3449 DEGs were detected in rice tissues. Surprisingly, 23 possible-candidate salinity responsive genes for yield and ion homeostasis were discovered.	[257]
	Mutation breeding	Rice mutants improve salt tolerance.	[119,121,123,124]
	Illumina-seq	DMRs enhance salt tolerance.	[131]
	Genetic engineering	Developed salinity tolerant rice mutants through CRISPR-cas9.	[258,259]
Transcriptomics	DNA microarray	486 salt-responsive ESTs identified from rice shoot.	[148]
	RNA-seq	Several salt-inducible genes have been identified	[152,153]
	RNA-seq	In hybrid rice LYP9 and from its two parents, salt- induced DEGs were found to be 8292, 8037 and 631, respectively. This research provided a new perspective on heterosis mechanisms in salinity tolerance.	[154]
	RNA-seq	More transporters, ion and sugar-related transports were also identified from Mulai roots to have a role in the control of salt tolerance.	[166]
	RNA-seq	Identify genetic SSR markers that will help in marker- assisted breeding to improve the agronomic traits under different stress conditions.	[163]
	RNA-seq	Identified important genes regulated during salt stress in rice, such as *OsSOS1*, *OsHKT1;5*, *OsHKT2;1*, *OsNHX1*, *OsAKT1*, *OsNRT1;2*, *OsTPC1*, *OsCDPK7*, *OsARP*, *OsMAPK5*, *44* and *OsSERF1.*	[76]
Proteomics	2-DE	Six salt responsive proteins identified	[181]
	2-DE and MALDI-TOF MS	During salt stress, 57 responsive proteins were regulated, among them several are novel salt responsive proteins.	[182]
	2-DE and LC-MS/MS	Four proteins were identified, among them 2 proteins, involved in salt stress response and the ubiquitin 26S proteasome system.	[183]
	2-D and MALDI-TOF MS	11 proteins were found to be differentially expressed. Most of them were new to being involved in rice salt response.	[185]
	2-DE	40 uniquely upregulated proteins were identified under ABA+salt stress.	[187]
	iTRAQ	Identified 5340 proteins, among them differentially expressed proteins involved in salt stress regulation and response to oxidation–reduction; photosynthesis and carbohydrate metabolisms.	[192]
	iTRAQ	Identified more than 2000 proteins in both root and shoot of salt-tolerant elite line FL478, during the early salinity stage. Among the identified proteins, some proteins are potential candidates, involved in the amino acid synthesis, antioxidant stress, and maintenance of mitochondrial activity, metabolism and Calvin cycle.	[193]
	iTRAQ	Identified 4598 proteins; among them, 279 were up- and downregulated and involved in oxidative phosphorylation, photosynthesis, phenylpropanoid biosynthesis, posttranslational modification and energy metabolism.	[194]
Metabolomics	GC-MS	Metabolic profiling of ice seeds.	[215,216,217,218]
	GC-MS	Rice metabolic profiling.	[219]
	H-NMR	Five conserved salts responsive metabolic markers were identified.	[220]
	H-NMR	Significant accumulation of sugar and amino acids under stress conditions.	[221]
	GC-MS	Characterised 92 primary metabolites in both shoots and roots in rice under stress and control conditions. Among them, 11 metabolites including amino acid and sugar significantly increased in tolerant varieties at the time of salt treatments.	[222]
	GC-MS	Two signalling molecules serotonin and gentisic acid are two significant biomarker compounds produced in tolerant varieties that contribute to NaCl tolerance	[224]
	GC-MS	A total of 84 metabolites were identified including amino acid, sugar, organic acid and other small molecular components.	[225]
Phenomics	RGB and fluorescence images	A combined technique was applied for the screening of different salt tolerance traits of rice.	[246]
	IR thermal images	Used to examine rice phenotyping under a salt stress environment.	[247]
	Automated imaging	Identify significant traits for subsequent QTL analysis, to deeper understand the genetic mechanisms driving RSA.	[248]
	X-ray tomography	Used to quantify the response of rice RSA to the soil environment.	[249]
	RGB and fluorescence images	Investigate the complex salinity tolerance in Australian wild rice species.	[251]

## 5. Modernise Breeding Approaches for Rice Salinity Improvement

**Figure 3:**
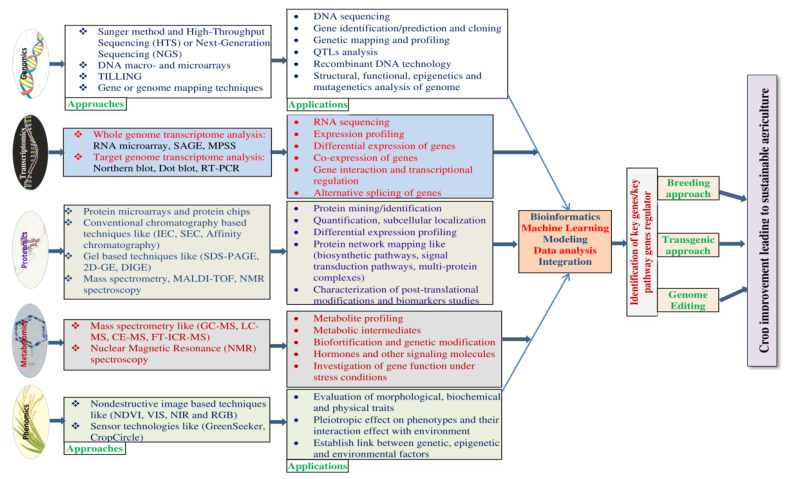
Schematic diagram of integrated omics for stress-tolerant rice improvement. To comprehend the complex features and to identify the key genes or regulators involved in salt tolerance, omics-based platforms should be merged. Essential genes need to be validated using functional genomic methods.

Game changing RNA sequencing, genotyping by sequencing, GWAS, and GEA analysis deposited a huge amount of biological data to integrate multi-omics with machine learning and user-friendly bioinformatics tools (Table 3), creating a new possibility to identify key genes involved in tolerance mechanisms by examining at interactions in metabolic pathways and network analysis to modernise the plant breeding such as Marker-Assisted Selection (MAS), transgenic approaches and genome editing (Figure 3) for crop improvement under abiotic stress conditions, including salinity.

### 5.1. Marker-Assisted Selection (MAS)

MAS, which involves the development of molecular markers linked to traits of interest has become a significant advance in stress biology, paving the way for accelerating rice breeding [16]. Due to the transfer of genomic regions of interest precisely, MAS became the most promising and very successful method for salt tolerance rice improvements [260]. This approach is independent of the growth stage of rice and is also unaffected by the environments [261]. Backcrossing is the most widely used technique for introgression or substitution of target genes or QTL from donor to recipient. Molecular markers, importantly SSRs and SNPs have been used to establish the backcrossing techniques. The Marker-Assisted Backcrossing (MABC) approach is a faster and more attractive tool for rice breeding and identifying genomic points of interest by using tightly linked molecular markers with agronomically important traits in rice as the foreground, background and recombinant selections for tolerance to abiotic stress, including salinity [13]. Saltol QTL was introduced via MABC in two different initiatives to improve salt tolerance export quality indica aromatic rice cultivars PB6 and PB1121. Saltol QTLs were also transferred into popular elite varieties in several countries through the MABC approach, such as Vietnam (cultivar AS996, BT7, Q5DB and Bacthom), Bangladesh (cultivar BR11 and BRRI dhan28) and West Africa (Rassi) [262,263,264,265,266]. However, very limited research has been undertaken to improve various abiotic stress tolerance rice varieties by MAS by pyramiding QTLs that influence tolerance to salinity, drought and submergence. A current study clearly indicated that yield and quality may be combined with major abiotic tolerance using a well-designed MABC approach assisted by minimal phenotypic selection [267]. Currently, marker-assisted gene pyramiding has been shown to be a promising strategy for developing salt-tolerant rice varieties.

### 5.2. Transgenic Approach

Rice faces various natural stresses such as drought, salinity, high temperature and cold, which affects the growth and yield of a plant [268]. Traditional breeders will not be able to obtain traits which are not inherent within the gene pool of their target plants through classical breeding. With recent improvements in the genetic engineering field, it is now possible to insert beneficial genes into a target plant, generating transgenic plants with multiple ideal traits [269]. By using genetic engineering, generation of transgenic plants resistant to abiotic stresses, especially salinity and drought, is most important in this “Global Warming’s Terrifying Era” [269]. Genetic transformation of rice by introducing beneficial traits to achieve desired gene expression is now a vital research technique in plant physiology and a practical tool for plant improvement [270]. Numerous types of plant transformation approaches are verified for stable introduction of foreign genes into the plant genome.

Transformation techniques can be categorised into indirect or direct gene transfer. Indirect gene transfer known as vector mediated gene transfer involves the introduction of exogenous DNA into the plant genome via biological vectors, whereas direct gene transfer involves the introduction of exogenous DNA directly into the plant genome via physical or chemical reactions [269]. *Agrobacterium*-mediated gene transformation involves tumour-inducing plasmid (Ti plasmid) based vector transformation and precise integration of a single copy number in the transgene into the plant genome [271]. This transient transformation is a good way to test gene function, promoter efficiency or the involvement of a protein in a short period of time [272]. These techniques are the most widely used and the best method for rice transformation for efficiency and effectiveness [269,273]. *Agrobacterium*-mediated transgenic plants create a new opportunity for crop improvement and plant gene functional research [274]. However, the indica subspecies of rice are the most difficult to regenerate, especially after transformation. Though, protocols for genetic transformation and regeneration of some major indica rice have also been published [274,275]. Regarding *Agrobacterium*-mediated transformation for monocotyledonous plant’s needs, many key factors must need to be considered such as germplasm, type and stage of explants, agrobacterium strain, vectors, acetosyringone, co-cultivation temperature and more efficient regeneration protocol.

To boost rice’s resistance to abiotic challenges, a set of abiotic stress-related genes has previously been introduced for the improvement of abiotic stress tolerance, including salinity. Previous studies demonstrated that transgenic rice harbour stress-related genes such as *NHX1* (vacuolar Na^+^/H^+^ antiporter) [276], *ADC* (arginine decarboxylase) [277], *Calcineurin*, *CBF3* (C-repeat element binding factor 3) [278], *codA* (choline oxidase) [279], *HVA1* (LEA protein) [280], *OsCDPK7* (regulatory factor) [49], *OsMAPK5* (mitogen-activated protein kinase) [281], *SOD2* (plasma membrane Na^+^/H^+^ antiporter) [282] and *TPSP* (trehalose-6-phosphate synthase and phosphatase) [283] to enhance abiotic stress tolerance, especially salinity and drought. A recent study also found that the overexpression salt-responsive gene *OsPP1a* [284], *OsASR1* [285], *Abp57* [286], *SIDP361* [287], *OsSUV3* [288] and *PDH45* [289] contribute to salinity tolerance in rice. As seen above, significant progress has been achieved in improving abiotic-stress-tolerant transgenic rice. However, no transgenic rice cultivar has been released for commercial cultivations. It is understood that abiotic stress, physiologically and genetically is very complex and influenced by sets of genes. Maker genes are also employed in transformation that may impact food safety and biosafety, limiting the usage of transgenic rice production. Genetically modified (GM) rice must follow the global Cartagena protocol, which is enforced in each nation by its own biosafety regulations [261]. Therefore, combining the transgenic techniques with traditional breeding and more advances in target specific gene editing technology will be a more suitable strategy to develop abiotic-stress-tolerant rice cultivars.

### 5.3. Genome Editing

Genetic engineering has evolved into a promising technique in modern plant breeding. Although physical and chemical mutagens cause random mutations, which have a limited mutation frequency in target loci, target mutations can be used in alternative mutagens [116]. Several systems have been used to target genome editing, such as meganucleases, zinc finger nucleases (ZFNs), transcription activator-like effector nucleases (TALENs) and clustered regularly interspaced short palindromic repeat (CRISPR). The use of the CRISPR/Cas9 system has accelerated rice functional genomic studies.

To date, CRISPR/Cas9 has proven to be the most powerful and most effective tool for rice salinity improvement due to its simplicity and high accuracy when compared to previous nuclease technologies such as TALENs and ZFNs [290]. The introduction of this technology has expanded the scope of agricultural research and provided chances to generate new plant varieties with novel traits for diverse abiotic stress situations along with combating diseases. CRISPR/Cas9 has already been used to modify rice genes for varietal improvement [291,292,293]. Until now, this technique has been successfully used in many eukaryotic species, including rice.

Studies have demonstrated that CRISPR/Cas9-mediated genome editing in rice genes, such as phytoene desaturase (*OsPDS*), betaine aldehyde dehydrogenase (*OsBADH2*), mitogen protein kinase (*OsMPK2*), alternative oxidase (*AOX1a*, *AOX1b*, *AOX1c*) and *RAV2* have a role in regulating the response to abiotic stress stimuli, including salinity [291,294,295]. Using CRISPR/Cas9 techniques, two rice protein kinase family genes (*SnRK2s*) were functionally identified and found to be positive regulators of salt stress tolerance [258]. A recent study reported that a salinity-tolerant mutant was developed via cas9 genome engineering targeting the rice gene *OsRR22* [259], implying that CRISPR/Cas9 is a highly effective tool for improving rice salinity. Moreover, functionally significant SNPs discovered in GWAS research can be used in genome editing. If GWAS is used to identify non-phenotypic rice variants such as eQTL, meQTL and mQTL, in addition to new and valuable alleles, genome editing can be used to simplify the combining of new alleles to revolutionise rice breeding, particularly genomics-driven crop design [296,297].

### 5.4. Machine Learning (ML)

In this post-genomic era, the rapid growth in high-throughput data has led to the development of remarkable techniques for obtaining a complete picture of how the mechanistic basis of plant response works from DNA sequences to multi-dimensional molecular phenotypes. Omics research involves not only acquiring molecular phenotypes, but also explaining them using sophisticated techniques. Recently, machine learning techniques have turned out to be exceptionally impactful in these tasks. Machine learning can be classified into two learning strategies, i.e., supervised and unsupervised, which are used to uncover useful information existing in the multi-omics resources [298].

Supervised learning is known for its ability to predict or classify new data by fitting a model to labelled training data that is either numeric (regression) or categorical (classification). The most common steps in supervised learning involve (i) fitting a model based on the experimental data, (ii) assessing the model and coordinating the parameters of the model, and (iii) designing the model and utilising it to predict the outcomes [18,299]. This supervised learning enables the model to connect the target variables (e.g., phenotype) with the knowledge hidden in the datasets (e.g., RNA-seq). The following are examples of supervised learning applications in plants, such as predicting high-yielding genotypes in soybean [300], predicting stress response gene in *Arabidopsis thaliana* [301], predicting long non-coding RNA (lncRNA) in various plants [302], and assigning the class of clementine varieties [303]. Unsupervised learning, on the other hand, identifies clusters of unknown samples using input feature variables with no specified outcome such as classes or groups [304]. Clustering is a common type of unsupervised learning. Unsupervised clustering has played an important function in classifying plants based on their taxonomy or function. For example, a study by Liu et al. [305] performed hierarchical clustering to understand the relationship between the plants and their metabolite content properties. Recently, unsupervised learning has also been used to prioritise active FBX genes with distinct functional activities in *A. thaliana* [306], selecting candidates of salt-responsive genes [307], and monitoring drought stress in affected areas of plantation [308].

As technology increases, a considerable amount of machine learning algorithms with higher estimation performance, such as K-nearest neighbour (KNN), support vector machine (SVM), random forest (RF), artificial neural network (ANN), probabilistic neural network (PNN), genomic random regression (GRR), convolutional neural network (CNN), deep belief network (DBN), multivariate Poisson deep learning (MPDL), multilayer perceptron (MLP), radial basis function (RBF) or generalised regression neural network (GRNN), are being reported to improve various stress tolerance mechanisms in plant research [103,309]. For example, the K-nearest neighbours (KNN) algorithm was adopted to identify salt-tolerant rice genotypes by developing models for non-destructive estimation of leaf ion content [310]. During stress adaptation, plants show visual symptoms at the leaf margins, including leaf drooping and wilting, which reduces chlorophyll content and impedes photosynthesis rate [311]. These physiological changes in leaves caused by stress were predicted using the SVM, RF and KNN, for precise estimation of leaves based on their morphological features at cellular levels [312]. Artificial neural networks (ANNs) such as MLP, RBF, and GRNN have also been reported to predict the morphological response of citrus to drought stress and concentration of micronutrients to banana yield. Both studies reported that the GRNN model, a genetic algorithm (GA) was revealed to be more promising than the MLP and RBF to determine the optimal conditions (i.e., levels of different factors or macronutrients) for achieving the best morphological features (i.e., stress response or high yield) [313]. GA is a prominent optimisation algorithm that creates a useful hybrid model when combined with ANN. This hybrid ANN-GA algorithm has been used widely in improving numerous agricultural systems such as crop adaptability to climate change [17], biogas production [314], and proliferate vegetative rootstock [315].

In another study, PNN was used to estimate the probability of maize and wheat belonging to the specific phenotypic class based on two input variables, including genomic and phenotypic data [313]. From the study, PNN outperformed the MLP algorithm in classifying respective crops to the correct phenotypic class and providing better classification in a balanced class of the continuous trait datasets [313]. By using the GRR algorithm, a genomic model was developed to predict the differential response of wheat to environmental stress through the genotype-by-environment interactions [316]. The estimation of plant stress due to nitrogen deficiency has also been investigated by quantifying stress levels that fuses image-based plant phenotyping and 23-layered CNN [317]. Multivariate Poisson deep learning (MPDL), which is built to capture signals in count data for genomic predictions, is one of the developing models that can be used by plant breeders for genotyping in order to understand the interaction between the dataset of genotypes and phenotypes [318]. Count data is commonly used in plant breeding as it allows for the measurement of phenotypic information such as the number of seeds, infected spikelets, or germination days to maturity [318,319]. The other type of machine model is known as DBN; however, its applications have been studied in tomato and pepper leaf disease classification [320], maize phenotype prediction [321], plant recognition-based image retrieval [322], and none of the studies on abiotic stress have used DBN thus far.

## 6. Integration of Omics and Role of Bioinformatics for Rice Improvement

Rice research faces several challenges, not only in terms of salinity but also in terms of all other abiotic stresses. A multigenic trait corresponds to the rice’s response to salt stress. It is not possible to understand the genetic complexity of rice under stress conditions at all levels with a single cutting-edge study. Omics, a modern biotechnological tool in rice improvement, can be used to study the genetic and cellular mechanisms underlying salt stress tolerance. Omics approaches have shown promise for adapting to salinity tolerance in rice, but with limited success. This demonstrates how omics approaches overlap and are interdependent.

Integrated omics is becoming more important in understanding the complex physiological, biochemical, and molecular insights of salinity tolerance in rice. Current advancements in omics platforms have resulted in a wealth of data, particularly in the area of complex traits. The genotype that determines phenotypic traits is referred to as “forward genetics”, whereas functional genomics based on expression patterns gives function to dominant candidate genes or loci. Proteomics and metabolomics were used to identify ultimate proteins and numerous metabolites synthesised in response to stress via various important metabolic pathways [323]. The outcomes of these networks allow for the plant phenotype to be identified under various stresses. Transcriptome analysis can be used to investigate the molecular basis of salt stress tolerance in rice and the genes activated by salt stress [324]. The technique is insufficient for uncovering the molecular mechanisms underlying salt-stress tolerance. High-quality genomics data and integrated omics analyses have been used to identify stress-responsive genes and proteins under different environmental stimuli. These analyses can help us understand plant metabolism and how plants respond to different environments [325]. Notable results in rice salinity improvement have been demonstrated by integrated omics application [136,227,228,229,326], and these multi-omics analyses have been used to determine distinctive salt tolerance pathways in other crops [327].

The integration of multi-omics approaches (such as genomics, transcriptomics, proteomics, metabolomics and phenomics) as shown in Figure 3 reveals pivotal roles in the identification of molecular regulatory networks, resulting in a holistic understanding of rice salt tolerance and reshaping rice breeding. Standardised databases and bioinformatics tools are required in this context to enable broad use of these vital resources.

Bioinformatics is defined as the study of organising and interpreting biological data using modern computational tools. It is a branch of biology that develops techniques and tools for extracting, analysing, integrating and visualising large amounts of biological data generated by omics approaches to better understand biological functions [328]. Omics platforms have provided a wealth of biological data and restored it into databases, which serve as a repository for markers, genes, various types of RNAs, proteins, metabolites and phenomics information of various crops. Bioinformatics is critical for every aspect of omics-based research in promoting rice breeding by managing and analysing diverse data to understand biological functions, which ultimately aids in the discovery of genes for various agronomic traits [328]. Several bioinformatics tools and databases have been developed to access omics databases and gather biological information. A few specialised resources have been established for multi-omics research in rice under various stress conditions (Table 3). It is imperative to develop a new bioinformatics tool that integrates data from all omics levels to strengthen future rice research.

## 7. Conclusions

Salinity is one of the major constraints on rice productivity worldwide. Due to saline water intrusion in coastal and adjacent areas, it is becoming more difficult to meet the growing demand for staple foods such as rice. Improper irrigation with moderately saline water in the rice field aggravated the situation. Plant breeders face a significant challenge in developing salinity-tolerant rice cultivars. Several approaches have been considered for modifying the genetic makeup of rice to confer high salinity with minimum yield loss. Rice demand is increasing in tandem with the world’s population growth.

Rice is a salt-sensitive cereal crop and is classified as a typical glycophyte. Salinity stress significantly affects rice at its morphological, physiological, biochemical and molecular levels. Due to its stress-polygenic nature, it has been extremely difficult to determine the exact mechanism of salinity in a particular genotype. The salinity mechanisms in rice are unknown. It is necessary to assimilate knowledge to fully comprehend the molecular mechanisms at the omics level of salt’s catastrophic effect on rice. The combination of recent omics, including genomics, transcriptomics, proteomics, metabolomics and phenomics, aids in the identification of genes/QTLs, proteins and metabolites involved in salt-stress tolerance. With the advent of NGS, increased availability of large data and integrated omics tools will be more helpful to identify major genes involved in stress tolerance mechanisms and introgressive genes to produce superior rice cultivars.

## 8. Future Directions

Several studies have demonstrated how multi-omics approaches can aid in the identification of stress-related candidate genes, proteins, metabolites and pathways. The focus of gene-based research has shifted from single genes to the whole genome, which helps researchers better understand genetic connections. The application of modern genomic techniques such as NGS improves the accuracy and efficiency of salinity-related QTL mapping. The advancement of low-cost, high-throughput technologies enables whole genome re-sequencing and molecular sequencing that aids in studying the genome and transcriptome of a population rather than an individual. On the other hand, advances in proteomics aid in more in-depth analysis such as membrane protein identification, post-translational modification and protein–protein interactions. One of the best options for better understanding the mechanisms of the salt stress response is organ-specific proteomic analysis combined with bioinformatics. Proteomics combined with other omics holds enormous promise for unravelling the complexities of stress response. More metabolites can be found in the future and used as biomarkers to investigate the stress tolerance response. Combining metabolomics with other omics can be an aid in understanding the distinct metabolic network involved in the biosynthesis process. The integration of genomic and metabolomic techniques facilitates the functional analysis of genes. Recent advancements in high-throughput phenotyping aid in the collection of high-dimensional phenotyping data at multiple levels. High-throughput phenotyping tools linked with genomics will be the most useful tools for identifying dynamic phenotypic traits. The integration of phenotypic data with genomics, transcriptomics, proteomics and metabolomics can be used to mine new genes/QTLs, thereby narrowing the phenotype–genotype gap. The key data mining strategies within the artificial intelligence framework have been rarely proposed until now, as well as novel integrative analyses that will assist in the near-future studies on omics functional prediction. Machine learning techniques may help to improve the prediction of abiotic stress tolerance by integrating heterogeneous datasets while sidestepping the curse of dimensionality. These findings may also aid in the discovery of salt stress mechanisms.

Diverse studies using omics tools and the integration of genomics, transcriptomics, proteomics, metabolomics and phenomics will be very promising avenues for identifying key underlying genetic and signalling networks that will help researchers understand the intricate links that exist between genes, proteins and metabolites and their biological activities within plants. As a result, multi-omics approaches will modernise traditional breeding programmes and expedite precision rice breeding through marker-aided gene pyramiding, genetic engineering and multiplex genome editing.

## Figures and Tables

**Table 3:** Online databases available for rice integrated omics analysis.

Database	Description	Web Tool/URL
RAP-DB	Rice genomics database	https://rapdb.dna.affrc.go.jp (accessed on 10 October 2021)
RiceXPro	Expression profile database of rice	https://ricexpro.dna.affrc.go.jp (accessed on 10 October 2021)
NCBI GEO	National Center for Biotechnology Information Gene Expression Omnibus	https://ncbi.nlm.nih.gov/geo (accessed on 10 October 2021)
QlicRice	Stress related QTLs data mining tool	https://nabg.iasri.res.in:8080/qlic-rice (accessed on 10 October 2021)
STIFDB2	Plant stress-related data mining tool	https://caps.ncbs.res.in/stifdb2 (accessed on 10 October 2021)
TENOR	Comprehensive mRNA-seq database of rice under environmental stress conditions	https://tenor.dna.affrc.go.jp (accessed on 10 October 2021)
Genevestgator	Transcriptomics database for investigating gene expression in a wide range of biological situations	https://genevestigator.com (accessed on 2 September 2021)
CSRDB	Small RNA database for cereals	https://sundarlab.ucdavis.edu/smrnas (accessed on 10 October 2021)
RiceSRTFDB	Rice stress-related TF database	https://nipgr.res.in/RiceSRTFDB (accessed on 10 October 2021)
Stress2TF	A manually curated database of transcription factor regulation in plants response to stress	https://csgenomics.ahau.edu.cn/Stress2TF (accessed on 10 October 2021)
PSPDB	Stress-related protein database for plants	https://bioclues.org/pspdb (accessed on 10 October 2021)
OryzaGenome	Integrated biological and genomics database	https://viewer.shigen.info/oryzagenome2detail (accessed on 15 October 2021)
Ricebase	Combining molecular marker, pedigree and whole-genome-based data tool	https://ricebase.org (accessed on 10 October 2021)
Gramene	A comprehensive data library for comparative genomics studies	https://gramene.org (accessed on 15 October 2021)
Phytozome	Plant Comparative Genomics Portal	https://phytozome.net (accessed on 12 February 2020)
Ensembl Plants	Integrated tool for plant genomics data mining, interpreting and visualising	https://plants.ensembl.org (accessed on 12 February 2020)
PlantPReS	Plant proteome database	https://proteome.ir (accessed on 17 October 2021)
Plant Reactome	Genome, transcriptome, proteome and integrated metabolic pathways	https://plants.reactome.org (accessed on 12 February 2020)
PlantGDB	Resources for plant genomics	https://plantgdb.org (accessed on 17 October 2021)
GabiPD	Integrative omics database	https://gabipd.org (accessed on 17 October 2021)
PMND	A vast network of databases on plant metabolic pathways	https://plantcyc.org (accessed on 17 October 2021)
RicyerDB	Integrated genomics and proteomics database	https://server.malab.cn/Ricyer (accessed on 13 October 2021)
CARMO	Integrative omics database	https://bioinfo.sibs.ac.cn/carmo (accessed on 13 October 2021)
PTools	Integrative omics database	https://omictools.com/ptools/tool (accessed on 13 October 2021)
Gromacs	Database of genomics, proteomics and metabolomics	https://omictools.com/gromacs/tool (accessed on 13 October 2021)
STRING	PPI network analysis containing functional association	https://string-db.org (accessed on 9 January 2020)
PANTHER	Analysis of proteins based on evolutionary relationships	https://pantherdb.org (accessed on 13 October 2021)

## Data Availability

Not applicable.
